# Impact of rising sea levels on Australian fur seals

**DOI:** 10.7717/peerj.5786

**Published:** 2018-10-16

**Authors:** Lachlan J. McLean, Steve George, Daniel Ierodiaconou, Roger J. Kirkwood, John P.Y. Arnould

**Affiliations:** 1 School of Life and Environmental Sciences, Deakin University, Burwood, Victoria, Australia; 2 National Centre for Atmospheric Science-Climate, University of Reading, Reading, United Kingdom; 3 School of Life and Environmental Sciences, Deakin University, Warrnambool, Victoria, Australia; 4 Research Department, Phillip Island Nature Parks, Cowes, Victoria, Australia

**Keywords:** *Arctocephalus pusillus*, Storm surge, Sea level, Pup mortality

## Abstract

Global warming is leading to many unprecedented changes in the ocean-climate system. Sea levels are rising at an increasing rate and are amplifying the impact of storm surges along coastlines. As variability in the timing and strength of storm surges has been shown to affect pup mortality in the Australian fur seal (*Arctocephalus pusillus doriferus*), there is a need to identify the potential impacts of increased sea level and storm surges on the breeding areas of this important marine predator in Bass Strait, south-eastern Australia. Using high-resolution aerial photography and topographic data, the present study assessed the impacts of future inundation levels on both current and potential breeding habitats at each colony. Inundation from storm surges, based on a predicted rise in sea level, was modeled at each colony from 2012 to 2100. As sea level increases, progressively less severe storm surge conditions will be required to exceed current inundation levels and, thus, have the potential for greater impacts on pup mortality at Australian fur seal colonies. The results of the present study indicate that by 2100, a 1-in-10 year storm will inundate more habitat on average than a present-day 1-in-100 year storm. The study highlights the site-specific nature of storm surge impacts, and in particular the importance of local colony topography and surrounding bathymetry with small, low-lying colonies impacted the most. An increased severity of storm surges will result in either an increase in pup mortality rates associated with storm surges, or the dispersal of individuals to higher ground and/or new colonies.

## Introduction

Global warming is leading to many unprecedented changes in the climate system that are impacting habitats and ecosystems (Cleugh et al., 2011; IPCC, 2014b). Acceleration in global warming can be largely attributed to anthropogenic activities releasing greenhouse gases into the atmosphere, leading to rising sea levels, and an increased frequency of extreme weather events (Cleugh et al., 2011; IPCC, 2014b). These impacts have the potential to alter the structure and function of marine ecosystems, leading to a decline in biodiversity (IPCC, 2014a).

Oceans play an integral role in the regulation of the global climate, absorbing one-third of the world’s carbon dioxide emissions and accounting for the majority of the world’s heat balance (Hoegh-Guldberg & Bruno, 2010; IPCC, 2014a; Levitus et al., 2000). Due to their size and complexity, the consequences of warming oceans are often poorly understood (Hobday et al., 2006; Hoegh-Guldberg & Bruno, 2010). However, the impact of thermal expansion on global sea levels is one consequence that has been well documented (IPCC, 2013).

Coastlines are the ecotone between marine and terrestrial environments (Ray, 1991). They support a high diversity of species that are specifically adapted to the unique environmental conditions (Burkett et al., 2008; Hobday et al., 2006). Islands provide marine mammals and seabirds with critical breeding habitats devoid of mainland predators (Baker, Littnan & Johnston, 2006). Impacts of storm surges and sea level rise on these critical habitats has the potential to cause declines in populations of marine mammals and seabirds (Dulvy, Sadovy & Reynolds, 2003). However, predicting the consequences can often be difficult due to the highly mobile nature and long life span of these species (Poloczanska et al., 2007; Simmonds & Isaac, 2007).

A storm surge is a temporary rise in water level created by intense surface winds and low atmospheric pressure, as defined by Lowe & Gregory (2005). Rising sea levels will accentuate the temporary extreme in sea level and wave height along coastlines (Lowe & Gregory, 2005). The severity of inundation from storm surges is unique to each coastal region: it is influenced by a number of oceanographic and terrestrial factors, such as topography, bathymetry, tidal fluctuations and local meteorology (Lowe & Gregory, 2005; Pugh, 2004). Concurrent ocean warming and sea level rise is altering the pattern and strength of winds and ocean currents, and increasing the frequency and intensity of storm surges (IPCC, 2007; Poloczanska et al., 2007).

Sea surface temperatures are projected to increase around Australia, with particularly large changes of up to 4 °C forecast along the east coast of Tasmania under a high emissions scenario in 2090 (CSIRO and Bureau of Meteorology, 2015; Lenton, McInnes & O’Grady, 2015). Projected warming in south-eastern Australia is largely due to the East Australian Current strengthening and delivering warmer waters further south into the Tasman Sea (Hobday et al., 2006; Poloczanska et al., 2007). Warmer waters in south-eastern Australia are predicted to influence the region’s marine flora and fauna, particularly in Bass Strait which hosts an abundance of endemic assemblages and important marine predator populations (Poloczanska et al., 2007; Pompa, Ehrlich & Ceballos, 2011).

**Figure 1:**
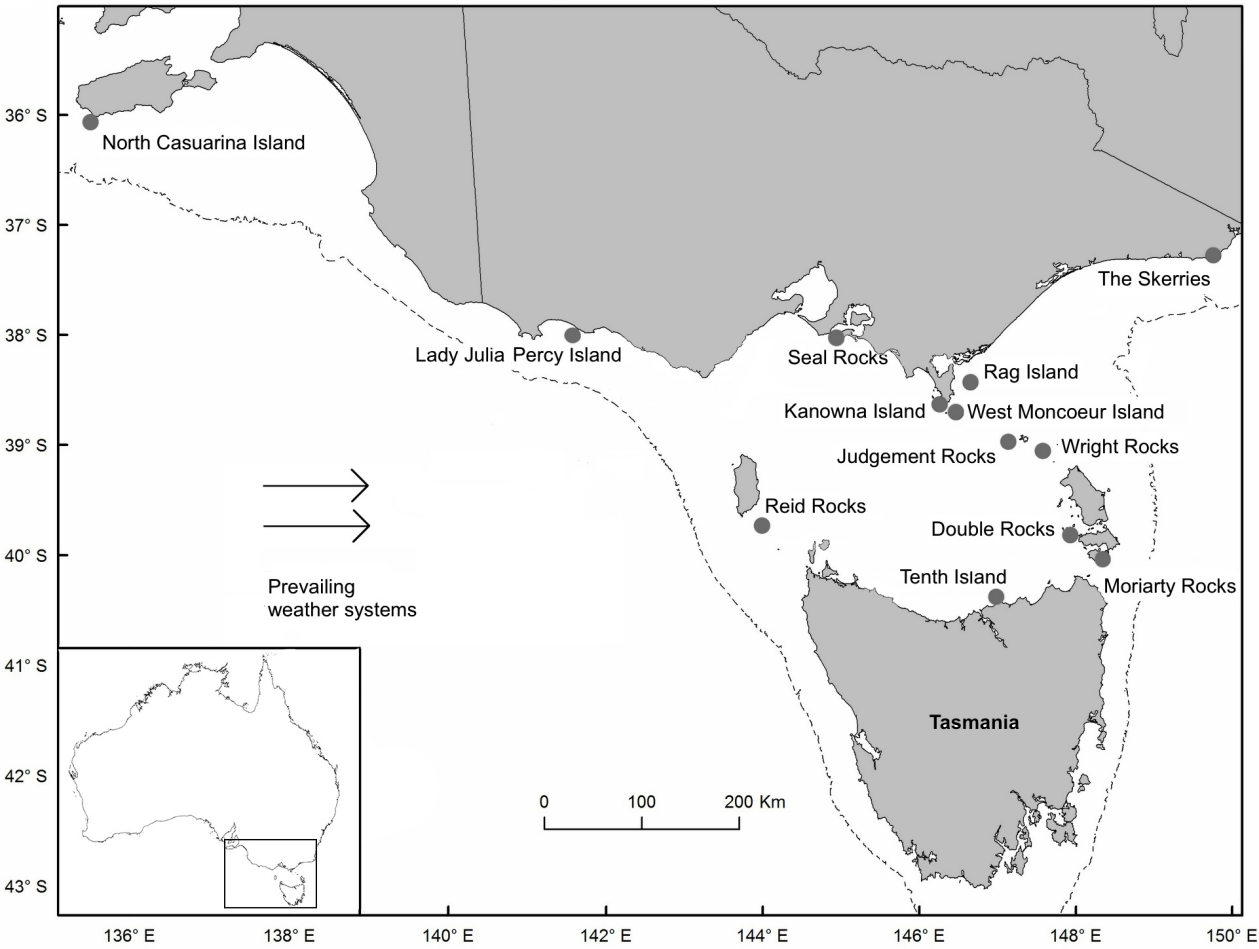
Location of Australian fur seal (*Arctocephalus pusillus doriferus*) breeding colonies in Bass Strait, south-eastern Australia. The continental shelf is outlined by the dotted 200 m bathymetric contour. Arrows indicate the direction of prevailing weather systems responsible for storm surges along the southeast coastline of Australia.

The Australian fur seal (*Arctocephalus pusillus doriferus*) is the prominent marine mammal in Bass Strait (Kirkwood & Arnould, 2012). With average female and male body masses of 76 kg and 279 kg respectively, and a current population size of *ca* 120,000 individuals, it represents the regions greatest predatory biomass (Kirkwood et al., 2010; Kirkwood & Arnould, 2012; Warneke & Shaughnessy, 1985). The species is recovering from over-exploitation and is currently one of the most geographically constrained fur seal species (Arnould & Kirkwood, 2007). The population is currently restricted to 13 breeding colonies within Bass Strait and its continental shelf approaches (Fig. 1), of which only two (Lady Julia Percy and Seal Rocks) account for ca 50% of the total annual pup production (Kirkwood et al., 2010).

Fur seals are one of the few groups of mammals that combine marine foraging and terrestrial parturition (Bartholomew, 1970). This amphibious lifestyle makes them vulnerable to both marine and terrestrial predators and, consequently, their breeding sites are largely restricted to off-shore islands (Riedman, 1990). These islands support the seals’ important life history stages including birth, nursing, mating and moulting (Warneke & Shaughnessy, 1985). The suitability of breeding habitat, in particular proximity to reliable foraging areas, influences the reproductive success of seals (Cassini, 2000; Kirkwood & Arnould, 2012).

Thermoregulation on land is particularly important for fur seals as their ability to cool when on land is impeded by dense fur, an adaptation to their life in temperate waters (Cassini, 2000; Limberger et al., 1986). Periods of high ambient temperatures can increase rates of pup mortality, highlighting the importance of colony attributes that aid thermoregulation, such as shade, rock pools and water access (Bradshaw et al., 1999). Direct contact with water is the principal thermoregulatory technique when coping with high air temperatures, resulting in colonies clustering at the water’s edge (Cassini, 2000; Trillmich & Trillmich, 1984). This clustering is particularly pronounced during the austral summer breeding season (November–December), when colonies are at their most populous and ambient temperatures are high (Cassini, 2000; Limberger et al., 1986; Pemberton & Gales, 2004; Shaughnessy, Kirkwood & Warneke, 2002).

During the early weeks of life, fur seal pups are weak and poor swimmers (Kirkwood et al., 2005) and individuals close to the water’s edge are susceptible to being washed off the colony by storm surges where they are vulnerable to drowning and starvation due to separation from their mothers (Mattlin, 1978; Trillmich & Trillmich, 1984). Therefore, storms that coincide with the breeding season can substantially reduce pup survival. As female Australian fur seals give birth to a single pup each year and have one of the lowest fecundity rates (53%) of any fur seal species (Gibbens, Parry & Arnould, 2010), a higher frequency of storm surges coinciding with the breeding season could cause population declines. However, the ability of the species to cope with increased storm surges will largely be determined by the local topography of each colony (Haigh et al., 2012; IPCC, 2012). Low-lying colonies are more exposed to large ocean swells and are likely to be more severely impacted by storm surge events than at sites with higher elevations. Low-lying colonies often show significant inter-annual fluctuations in pup numbers, with >50% pup mortality experienced during years of severe storms during the pupping period (Kirkwood et al., 2005; Pemberton & Gales, 2004; Pemberton & Kirkwood, 1994). A predicted increase in the frequency and severity of storm surges (McInnes et al., 2009) during the breeding season may therefore have dramatic consequences for pup survival and recruitment.

The ability of the species to adapt to these impacts will be influenced by many factors, including high site fidelity, and the availability of prey resources within the foraging range (Kirkwood & Arnould, 2012; Schumann et al., 2012). Understanding these impacts is crucial for the future management of this top order marine predator (Goldsworthy et al., 2003). However, there is currently a paucity of data regarding the vulnerability of Australian fur seal colonies to storm surges (Schumann et al., 2012).

The aims of this study, therefore, were to: (1) assess the current and potential breeding habitat at Australian fur seal colonies; (2) predict how each site will be impacted by sea level rise and storm surges under future climate change scenarios; and (3) combine these data to estimate future impacts of sea level rise and storm surges on Australian fur seal status and distribution.

## Materials and Methods

### Current and potential breeding habitat

To aid in the identification of breeding areas, aerial photographs of the highest available resolution were obtained for 11 Australian fur seal colonies in Tasmania and Victoria (Table 1). These sites represented the breeding area for approximately 99% of the Australian fur seal population in 2007 (Kirkwood et al., 2010). Aerial photographs were acquired through the Tasmanian Department of Primary Industries, Parks, Water and Environment (DPIPWE), Phillip Island Nature Parks (PINP) and Google Inc. (Google Earth). Seal colony habitats were identifiable by the presence of seals, as well as the distinctive light substrate colour resulting from years of deposition of seal faeces and stains from pelage-oil (Bradshaw et al., 1999; Pemberton & Gales, 2004). Current breeding habitats were further refined with knowledge from visits to colonies (by R Kirkwood and JPY Arnould).

**Table 1:** Summary of data collected on Australian fur seal (*Arctocephalus pusillus doriferus*) breeding colonies in Bass Strait, south-eastern Australia.

**Colony**	**Aerial photography**	**Topographic data**	**Topographic data format**
Tenth Island	DPIPWE	DPIPWE	1 m contours
West Moncoeur Rocks	DPIPWE	DPIPWE	10 m contours
Judgement Rocks	DPIPWE	DPIPWE	10 m contours
Wright Rocks	DPIPWE	Marine World Database	Maximal height
Moriarty Rocks	DPIPWE	Marine World Database	Maximal height
Seal Rocks	PINP	PINP	LiDAR
Rag Island	Google Earth Satellite Imagery	Google Earth	Maximal height
The Skerries	Google Earth Satellite Imagery	DSE	LiDAR
Reid Rocks	DPIPWE	Marine World Database	Maximal height
Lady Julia Percy Island	Google Earth Satellite Imagery	Marine World Database	Maximal height
Kanowna Island	Google Earth Satellite Imagery	Deakin University	Point data

Aerial photographs were georeferenced in ArcGIS 10.2 (Esri, Redlands, CA, USA) by aligning them with ground control points (Calzadilla Pérez et al., 2002). High resolution aerial imagery obtained was georeferenced to coarse resolution base maps from Google Earth using identifiable features in both sets of imagery under the Geocentric Datum of Australia 1994 projection. For two Tasmanian colonies, Moriarty Rocks and Wright Rocks, there was no Google Earth imagery available, therefore, tie points were obtained from a Draft Management Plan for the Small Bass Strait Island Reserves (Parks and Wildlife Service, 2000) and Google Inc. Maps.

Topographic data was overlayed on aerial photographs to provide a digital terrain model of each island. Topographic data was sourced from the Tasmanian DPIPWE, Deakin University, the Victorian Department of Sustainability and Environment (DSE), Phillip Island Nature Parks, Google Inc. (Google Earth) and the Marine World Database (Newsome & Newsome, 2009) (Table 1). Topographical resolution ranged from maximal heights (m) for rocky outcrops to full coverage digital terrain models from Light Detection and Ranging (LiDAR) imagery.

Spot elevation data from field studies at Kanowna Island was converted to a raster layer in ArcGIS 10.2 using the Topo to Raster tool in the Spatial Analyst, Interpolation toolset (Esri, Redlands, CA, USA). From this, contours were extrapolated to 10 cm intervals using the Contour tool in the Spatial Analyst, Surface toolset. At colonies where only a single maximum height was available, contours were digitised using aerial photographs and knowledge from site visits. For colonies where topography was extrapolated, the low water mark was manually delineated using aerial photographs digitising the land-ocean boundary and thus making an assumption images were captured at low tide to avoid overestimating the extent of breeding habitat.

Using existing breeding habitats and local topography of sites as a guide, potentially suitable breeding habitats were estimated based on accessibility of unexploited areas. Pup densities were calculated by dividing current breeding areas (m^2^) by estimates of live pup numbers recorded in 2007 by Kirkwood et al. (2010).

### Modeling of sea level rise and storm surge

**Figure 2:**
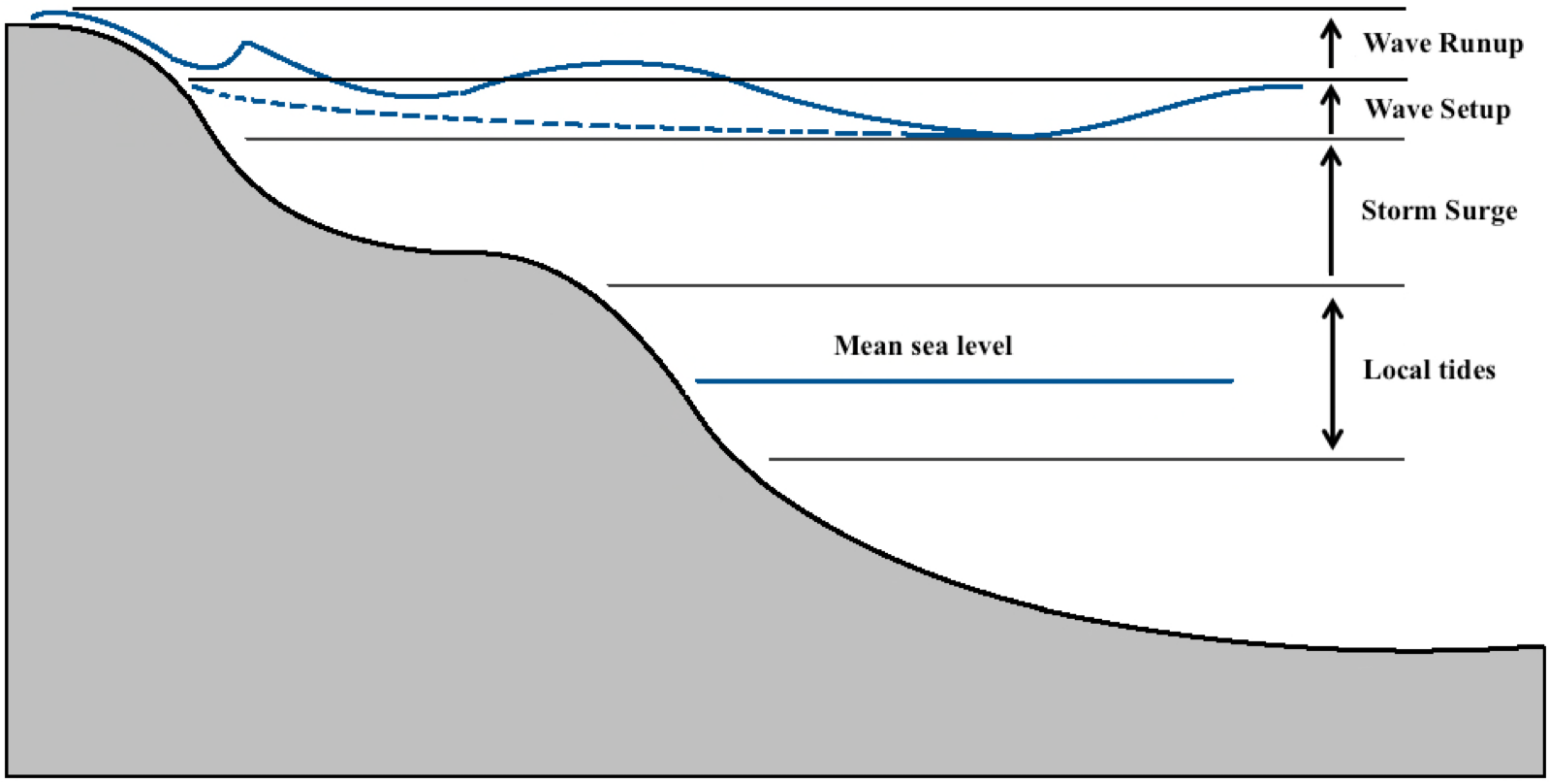
Schematic of contributions to maximum coastal sea levels modified from McInnes et al. (2012). Over time the earth will deviate from the current mean sea level due to climate induced sea level rise. Storm surges caused by high winds and variations in atmospheric pressure will cause temporary increases in sea level. Wave setup is a result of increased mean water level at shore due to the forward momentum of waves. Wave runup is the broken swash of the wave pushing up the beach to create a maximal height above sea level. The impacts of increased levels are most exacerbated during periods of high tide.

The impact of inundation at colonies is influenced by sea level rise, tidal range, storm surge, wave setup and wave runup (Fig. 2). Present-day and future inundation levels of colonies were calculated relative to mean sea level defined by the Australian Height Datum (AHD).

Future extremes in sea level were estimated using Canute 2.0 model, developed by Hunter (2010). Canute 2.0 combines present-day extreme sea levels with projections of future mean sea level rise. Present-day extreme sea levels are estimated by reconstructing historical sea level, storm surge and tidal data over a period of 61 years (Haigh et al., 2014). Predictions were confined to the November–December seal breeding season in Bass Strait.

The Canute 2.0 model simulates extremes in sea level arising from contributions from mean sea level, astronomical tides and storm surges (Haigh et al., 2014). Storm surge components were calculated using historical meteorological data, including sea level pressure fields and wind vectors (Haigh et al., 2014). Present-day extremes in sea level were then combined with projections of sea level rise over the next century.

Projections of sea level rise were provided for three future emission pathways: A1FI (fossil fuel intensive); A1B (balanced energy sources); and B1 (a world more integrated and ecologically aware) (IPCC, 2000). In 2014 these scenarios were superseded by four Representative Concentration Pathways (RCPs) in the Fifth Assessment Report of the IPCC (2014b). Each RCP defines an emissions pathway for the 21st century and together they represent a wider range or scenarios than the Special Report on Emissions Scenarios (SRES) used previously (IPCC, 2007; IPCC, 2014b). RCP8.5 is broadly comparable with the A1FI scenario, but projects a slightly higher rise in global mean sea level (IPCC, 2007; IPCC, 2014b).

Currently, greenhouse gas emissions and sea levels are following the fossil fuel intensive A1FI and RCP8.5 scenarios (IPCC, 2014b; Rahmstorf et al., 2007). Subsequently, organisations such as the Australian Federal Government’s National Climate Change Adaptation Research Facility are adopting these into their modeling scenarios (Hunter, 2010; McInnes et al., 2015; Woodroffe et al., 2012). Due to the present-day trends of greenhouse gas emissions, the current study focuses on the A1FI scenario.

Results from the Canute 2.0 model were combined with impacts of total wave runup (wave setup and runup). Total wave runup was modeled using the Antarctic Climate and Ecosystem Cooperative Research Centers (ACECRC) online calculator, based on research by Morang & Szuwalski (2003) and the Federal Emergency Management Agency (2003). The ACECRC’s online model required inputs of significant wave height (Hs), peak wave period (Tp) and beach gradients at each colony. Hs defines the average of the highest one-third of waves over a given time (Morang & Szuwalski, 2003). Tp defines the period, in seconds, between each wave’s point of highest energy (Morang & Szuwalski, 2003).

Mean values of Hs and Tp for November and December were produced using the National Oceanic and Atmospheric Administration’s WAVEWATCH II model. Values were modeled from 22 years of historical data collected at each colony’s nearest coastal data point (on mainland Australia and Tasmania). Beach gradients were calculated by determining the elevation to distance ratio between colonies’ surrounding bathymetry and coastal topography in ArcGIS 10.2 (Esri, Redlands, CA, USA). In a recent effort to assess the impacts of climate change on the coastal zone, the Department of Sustainability and Environment of the Victorian State Government commissioned and made available a high-resolution LiDAR dataset covering the Victorian coastline. This dataset includes seamless terrestrial-marine mosaics from elevations of +10 m to depths of −20 m, for a surface coverage exceeding 10,000 km^2^ (Quadros & Rigby, 2010). Bathymetric data used to calculate wave setup and runup were based on bathymetric LiDAR contours at 5 m intervals. Gradients were calculated on the west side of each colony, this being the prevailing wind direction in Bass Strait during storm surge events (McInnes et al., 2013).

Total inundation levels were calculated on a scale of centimetres. In order to model inundation levels, each colony’s topography was interpolated to 10 cm contours. Inundation levels were predicted for 10, 50 and 100 year average recurrence intervals (ARI). ARIs are an estimate of the time between predicted storm surges and are used to express the likelihood of extreme events (Haigh et al., 2012). A storm surge with an ARI of 10 years has a 10% probability of occurring each year. Similarly, an ARI of 100 years has a 1% probability of occurring each year. Inundation levels for each ARI were predicted for 2012, and 2100.

### Potential impacts of future inundation

Predicted inundation levels were modeled at each colony to estimate the impacts of habitat inundation. Impacts of regular events are more likely to elicit a species’ response as opposed to stochastic events (Dere et al., 2006). For example, Australian fur seals will breed in areas that are usually safe from ‘normal’ levels of inundation, and will get ‘caught out’ by abnormal events. Accordingly, this study focused on the impacts 1-in-10 year storm surge events.

**Figure 3:**
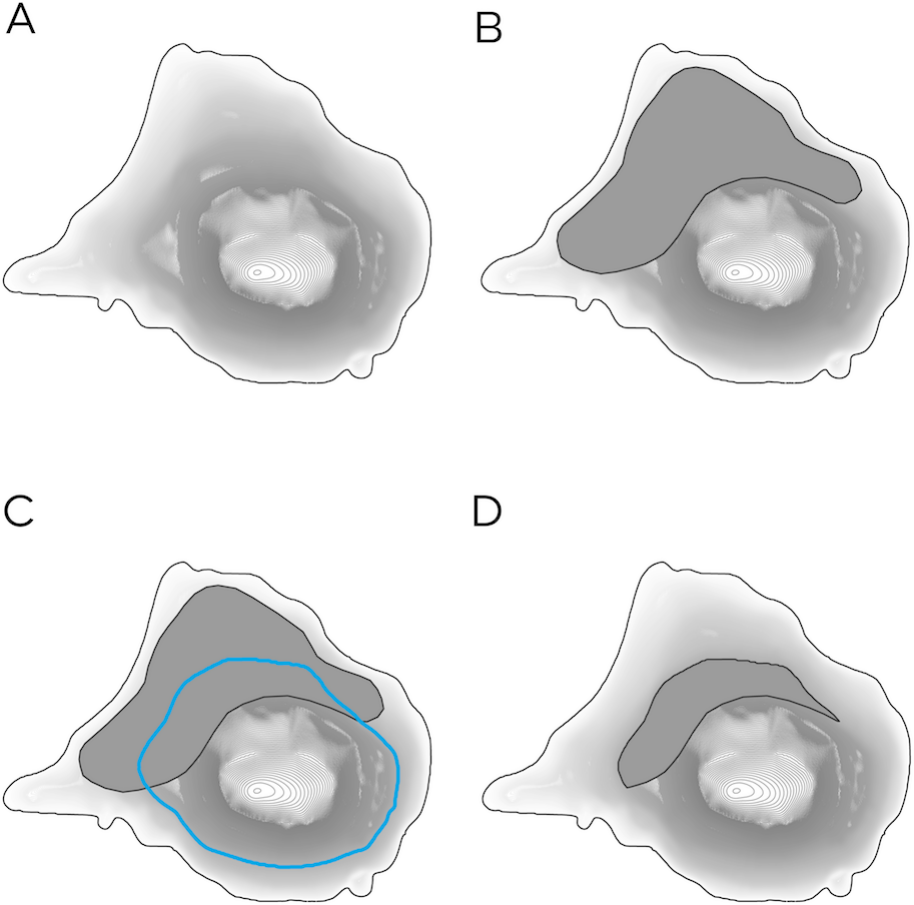
Calculating the impacts of predicted inundation levels at a hypothetical seal colony. (A) The island’s topography is established. (B) Breeding habitat (dark grey polygon) is identified and overlain. (C) The predicted inundation level (blue contour) is derived from the colony’s topography. (D) Breeding habitat below the predicted inundation level is removed leaving only suitable habitat above the predicted inundation level.

Current and potential breeding habitats were then reduced to habitat above future inundation levels (Fig. 3). To minimise overestimation of impacts, inundation levels were rounded down to the nearest 10 cm contour line. The area of the reduced habitat was then used to calculate impacts of habitat loss. Reduced habitat was divided by current habitat, to provide a percentage of habitat remaining. From this, the percentage of habitat loss could be calculated.

## Results

### Colony characteristics

Australian fur seal colonies occur almost exclusively on islands, which vary from >1 km^2^ vegetated islands (Kanowna Island) to small rocky outcrops (The Skerries). These islands range in elevation from 7 to 104 m above sea level (Table 2). Breeding habitats and densities of seals during breeding season also vary greatly between colonies. Current breeding habitat sizes range from 1,438 to 47,108 m^2^. Maximum densities of pups in these habitats were estimated to range from 0.02 to 0.59 pup m^−2^.

**Table 2:** Summary of Australian fur seal (*Arctocephalus pusillus doriferus*) breeding colony features.

**Colony**	**Maximum elevation (m)**	Current breeding area (m^2^)	**Pup production**	Density (pup m^−2^)	**Potential breeding area** (m^2^)
Tenth Island	10	1,439	530	0.37	1,439
West Moncoeur Rocks	90	3,735	240	0.06	3,735
Judgement Rocks	30	5,685	2,800	0.49	8,146
Wright Rocks	40	8,414	150	0.02	26,509
Moriarty Rocks	7.6	9,692	700	0.07	9,692
Seal Rocks	13.25	11,353	6,700	0.59	22,727
Rag Island	18	13,188	330	0.03	62,694
The Skerries	7.1	18,190	3,200	0.18	18,190
Reid Rocks	12	22,787	1,000	0.04	22,787
Lady Julia Percy Island	53	42,305	6,600	0.16	1,244,180
Kanowna Island	104	47,108	3,400	0.07	231,089

Many of the low-lying colonies are limited in available habitat, and therefore seals already occupy all areas. For example, Tenth Island (off the north coast of Tasmania) is the smallest colony in terms of area and all areas are occupied by the seals. Consequently, seals use the colony’s entire potential habitat. At larger, more elevated islands seals use only a small portion of the available habitat. For example, Lady Julia Percy Island hosts one of the largest Australian fur seal breeding colonies, with an estimated 30,000 total seals recorded in 2007 (Kirkwood et al., 2010). At present, the seals occupy small areas at the base of cliffs around the islands edge in coves, bays and rocky platforms (Shaughnessy, Kirkwood & Warneke, 2002). The current breeding area totals 42,305 m^2^ of a potential extent of 1,244,180 m^2^, including a large plateau on the top of the island, which has limited accessibility for seals (Table 2).

### Colony inundation levels

Over the next century, a rise in sea level will result in increased inundation levels across all colonies. Results showed an expected increase in inundation levels between 1-in-10 year and 1-in-100 year storm surges (Table 3). Increases will be comparable across all colonies, averaging 0.63 ± 0.02 m. By 2100, as a result of sea level rise, 1-in-10 year storms are predicted to have a greater impact than present day 1-in-100 year storms (Table 3). This increase in sea level will also impact higher frequency events such as yearly storms.

**Table 3:** Present-day (2012) and future predicted (2100) inundation levels from storm surge and sea level rise at Australian fur seal (*Arctocephalus pusillus doriferus*) breeding colonies in Bass Strait. Inundation levels are displayed for 1-in-10, 1-in-50 and 1-in-100 year storms.

**Colony**	**2012**			**2100**		
	*1-in-10 (m)*	*1-in-50 (m)*	*1-in-100 (m)*	*1-in-10 (m)*	*1-in-50 (m)*	*1-in-100 (m)*
Tenth Island	1.61	1.73	1.79	2.22	2.38	2.43
West Moncoeur Rocks	1.44	1.57	1.63	2.05	2.21	2.27
Judgement Rocks	1.44	1.57	1.63	2.05	2.21	2.27
Wright Rocks	1.36	1.48	1.53	1.98	2.13	2.18
Moriarty Rocks	1.15	1.26	1.31	1.77	1.92	1.97
Seal Rocks	1.65	1.78	1.84	2.25	2.41	2.48
Rag Island	1.33	1.45	1.50	1.95	2.10	2.15
The Skerries	1.20	1.31	1.36	1.81	1.96	2.02
Reid Rocks	1.19	1.31	1.35	1.81	1.96	2.01
Lady Julia Percy Island	0.76	0.87	0.92	1.37	1.52	1.58
Kanowna Island	1.55	1.68	1.74	2.15	2.31	2.37

Results highlighted variation in predicted inundation levels between colonies. During a future 1-in-10 year storm, for example, predicted inundation levels ranged from 1.37 m at Lady Julia Percy Island, to 2.25 m at Seal Rocks (Table 3). Greater inundation levels were predicted for colonies located in central Bass Strait, than for colonies located on the western and eastern outskirts of Bass Strait (Fig. 1).

Wave setup and runup values were reconstructed from historical data, independent of any increase in the frequency or intensity of storm surge activity (Table 4). Impacts of wave setup and runup were combined with predicted sea level rise and storm surge values to provide predictions of total inundation levels. Wave setup and runup values were the largest contributing factor to total inundation levels and had the greatest influence on inter-colony variation (Table 4).

**Table 4:** Contributing factors to total wave runup (wave setup and wave runup) at Australian fur seal (*Arctocephalus pusillus doriferus*) breeding colonies in Bass Strait, south-eastern Australia. Contributing factors include significant wave height (*H*_*s*_), peak wave period (*T*_*p*_) and beach gradient, which are modelled to provide total wave runup.

**Colony**	*H*_*s*_**(m)**	*T*_*p*_**(s)**	**Beach gradient**	**Total wave runup (m)**
Tenth Island	0.99	4.94	0.04	1.10
West Moncoeur Island	1.70	10.05	0.18	7.10
Judgement Rocks	1.70	10.05	0.14	6.00
Wright Rocks	1.70	10.05	0.16	6.50
Moriarty Rocks	1.54	7.54	0.02	1.40
Seal Rocks	2.08	11.39	0.08	5.10
Rag Island	1.70	10.05	0.16	6.50
The Skerries	1.89	7.72	0.03	2.00
Reid Rocks	0.99	4.94	0.31	4.30
Lady Julia Percy	3.07	12.20	0.08	6.90
Kanowna Island	1.70	10.05	0.38	11.80

### Impacts of inundation at colonies

The predicted areas of inundation varied largely due to each colony’s surrounding bathymetry and coastal topography. Hs and Tp also influenced the severity of inundation levels and varied greatly between colonies (Table 4). Total wave runup values were largest at Kanowna Island, which had large Hs and Tp, values, as well as a steep beach gradient. In contrast, Tenth Island had the smallest total wave runup value due to relatively small Hs and Tp values.

Larger, more elevated colonies such as Kanowna Island show a minimal increase in habitat inundation of 1%. In contrast, Tenth Island is a relatively small colony and shows a 25% increase in habitat inundation (Table 5). These varying rates of increase lead to a large range in the percentage of habitat inundated by 2100. Current breeding habitat inundated during a future 1-in-10 year storm surge ranges from 29 to 100% and the inundation of potential breeding habitat ranges from 3 to 92%.

**Table 5:** Total inundation levels and percentage of current and potential breeding habitat inundated at Australian fur seal (*Arctocephalus pusillus doriferus*) breeding colonies during a 1-in-10 year storm.

**Colony**	**2012**			**2100**		
	Total inundation (m)	Current habitat (%)	Potential habitat (%)	Total inundation (m)	Current habitat (%)	Potential habitat (%)
Tenth Island	2.71	37	37	3.32	62	62
West Moncoeur Rocks	8.54	64	64	9.15	67	67
Judgement Rocks	7.44	81	66	8.05	85	71
Wright Rocks	7.86	55	36	8.48	61	40
Moriarty Rocks	2.55	72	72	3.17	83	83
Seal Rocks	6.75	100	78	7.35	100	81
Rag Island	7.83	74	29	8.45	79	32
The Skerries	3.20	85	85	3.80	92	92
Reid Rocks	5.49	11	11	6.11	33	33
Lady Julia Percy	7.66	85	3	8.27	87	3
Kanowna Island	13.35	28	6	13.95	29	6

## Discussion

Australian fur seal colonies are established predominantly on low-lying areas of rocky islands, where variability in the timing and strength of storm surges already causes substantial inter-annual variability in pup mortality. Through generations of trial, the seals have colonised these sites as they afford the best habitat for pup survival, provide access to water for thermoregulatory requirements and are in close proximity to suitable feeding areas. Climate change over the next 100 years will lead to increased sea levels and an increase in the severity of water levels associated with storm surge events over southern Australia (Colberg & McInnes, 2012). This will result in more frequent inundation at Australian fur seal colonies, increasing pup mortality rates and/or leading to colonisation of new breeding sites.

This study highlights the fine-scale spatial variability of the impacts of global warming and emphasises the importance of wave setup and runup. Wave setup and runup will contribute more to the total area of inundation than sea level rise and storm inundation alone. This study also predicts where the greatest impacts of increased inundation will occur for Australian fur seals. Results may direct future management and understanding of changes to the overall distribution of this fur seal, which already has one of the smallest geographic ranges and populations of any fur seal species.

### Inter-colony variation in inundation

Inundation modelling at Australian fur seal colonies for the next century identified that the greatest inundation levels would occur at colonies most distant from the edge of the continental shelf, i.e., in the central Bass Strait and along the central Victorian and central-northern Tasmanian coastlines. The smallest levels of inundation occurred at Lady Julia Percy Island, Moriarty Rocks, Reid Rocks and The Skerries, islands all located on the outskirts of Bass Strait. This aligns with research by McInnes et al. (2009) and McInnes et al. (2012) who identified storm surges to be greatest in central Bass Strait from Port Phillip Bay to the north coast of Tasmania. This is due to the prevailing westerly cold fronts associated with storm surges in the south-east Australian region (McInnes et al., 2013). Storm surge impacts are amplified in central Bass Strait due to the larger tidal range, resulting in greater impacts than at outer-lying colonies (McInnes et al., 2012).

Coastal orientation and position can also influence the severity of impacts with coastal features such as headlands and bays providing more shelter at some colonies (Haigh et al., 2012). For example, although Rag Island is near Kanowna Island and West Moncoeur Island adjacent to the central Victorian coast, predicted inundation levels were lower there than the other islands. This may be due to the shelter provided by Wilsons Promontory, located west of Rag Island, during storm surges associated with westerly cold fronts.

Unlike storm surge and sea level rise impacts, wave setup and runup is largely influenced by surrounding bathymetry and coastal topography, which determine how wave energy is dissipated as it approaches shore. For example, if the bathymetric and topographic gradient is gradual, wave energy dissipates over a longer distance. However, if the gradient is short and steep, the wave energy will be stopped abruptly, resulting in larger wave runup impacts.

The largest total wave runup value occurred at Kanowna Island off the tip of Wilsons Promontory. This can be attributed to large Hs and Tp values, which are amplified by the steep gradients associated with the island’s coastline and surrounding sea floor, which is typical of granitic coast outcrops in this region (Kennedy, Ierodiaconou & Schimel, 2014). In contrast, Tenth Island is a low-lying rocky outcrop surrounded by a very gradual sea floor gradient. The colony’s gentle gradient, combined with minor Hs and Tp, results in the smallest total wave runup impact. However, the overriding influence on the severity of impacts was colony’s size and elevation, with small, low-lying colonies impacted the most.

### Inundation impacts

Modeling predictions indicate an increase in storm surge inundation levels driven by a predicted rise in mean sea level. With increasing sea levels, colonies will require progressively less severe storm surge conditions to exceed current inundation levels. For example, by 2100, a 1-in-10 year storm will inundate more habitat than a current 1-in-100 year storm. These trends are consistent with McInnes et al. (2012) and McInnes et al. (2013), who showed small increases in mean sea level resulting in relatively large increases in the frequency of storm surge events in south-eastern Australia.

Current model predictions of inundation levels are likely to underestimate future impacts. Predicted increases in inundation outlined in this study are based on increases in mean sea level, and do not include potential increases in the intensity or frequency of extreme weather events, such as 1-in-10 year storm surges (Hunter, 2010; IPCC, 2012; Schumann et al., 2012). The study also uses mean values of Hs and Tp over the November and December breeding season and does not account for any dependency between Hs and Tp during storm surge events. The inclusion of additional data on storm surge characteristics and their influence on mean Hs and Tp values would increase the accuracy, and potentially the severity of future inundation levels. Haigh et al. (2012), however, indicated that increases in mean sea level will be the dominate driver of future inundation levels in Australia, and changes in storm patterns will be comparatively small.

The impact of habitat loss is largely dependent on the size and elevation of breeding habitat. For example, the majority of breeding habitat at Kanowna Island occurs 20 m above sea level and totals almost five hectares. An increase of 0.6 m inundation reduced the colony’s current breeding habitat by only 1%. In contrast, breeding habitat at Tenth Island occurs 0.5 m above mean sea level and totals only 0.14 hectares. An increase of 0.61 m inundation decreased the current breeding habitat by 25%.

As sea levels rise, the frequency and extent of habitat inundated during storm surges will increase, leading to an increased frequency of pup mortality events. This will impact on juvenile recruitment and reduce the likelihood of recovery between events (Harley et al., 2006; Schumann et al., 2012). Currently, the average neonatal mortality in Australian fur seal pups is estimated at 13–15% (Pemberton & Kirkwood, 1994; Warneke, 1975). Starvation, injury, infection and drowning are the major causes of early pup mortality (Warneke, 1975). Larger fluctuations in pup mortality have been recorded in association with large storm surge events (Pemberton & Gales, 2004). This is particularly prevalent at low-lying colonies where pup counts have fluctuated up to 86% between years and personal observations have recounted seeing pups beached or in the water up to 50 km away following storm surge events during the breeding season (Pemberton & Gales, 2004; Pemberton & Kirkwood, 1994).

### Potential response to increased inundation events

The Australian fur seal population is still recovering from over harvesting during the 18th and 19th centuries, and at *ca* 120,000 individuals, it is currently only at 50% of its estimated maximum pre-exploitation level (Kirkwood et al., 2010; Pemberton & Gales, 2004; Warneke & Shaughnessy, 1985). Consequently, many of the current colonies may not have reached their carrying capacity, with other sites in Bass Strait yet to be recolonised. While some of the current breeding habitats are subject to periodic storm activity, the modelled increases in inundation levels potentially will inhibit the population’s recovery to pre-exploitation levels.

In the face of an increased severity of storm surges, individuals at colonies may respond by either: (1) continuing to breed in areas that are becoming increasingly inundated by storm surges, potentially causing higher pup mortality rates and impacting on the colony’s long-term demographics; (2) retreating to areas higher or further from the coast to avoid inundation, leading to higher operational breeding densities; and/or (3) moving to other colonies or colonising new areas.

Many fur seal species display a high degree of natal site fidelity (Campbell et al., 2008; Hoffman, Trathan & Amos, 2006) , which may influence whether or not individuals continue to breed in current areas even in the face of increasing storm surge events. If individuals remain in current breeding habitats, colonies will experience an increase in storm surge associated pup mortality. Sustained high levels of pup mortality will reduce juvenile recruitment resulting in an ageing and ultimately declining population (Craig & Ragen, 1999; Frederiksen et al., 2008).

**Figure 4:**
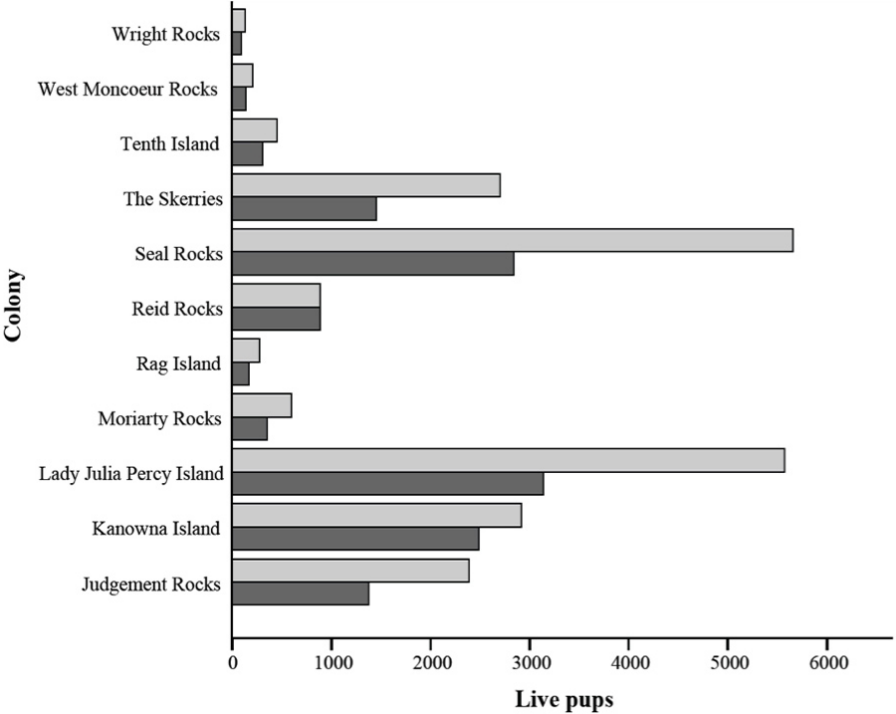
Comparison of current live pup estimates from Kirkwood et al. (2010) and 2100 live pup estimates. Current live pup estimates are represented by light grey bars. 2100 live pup estimates are represented by dark grey bars and are based on a 50% mortality rate of pups occurring in areas inundated by a 1-in-10 year storm, assuming current breeding distributions are maintained.

Based on an estimated pup mortality rate of 50% experienced in some years of large storm surges (Pemberton & Gales, 2004), a potential reduction in pup production can be estimated using the percentage of habitat inundated by a 1-in-10 year storm in 2100 (Fig. 4). Impacts will be particularly detrimental at colonies such as Seal Rocks, which supports a large portion of the species total pups born (26%), compared to Wright Rocks, which supports only a small portion of the total pups born (1%) (Kirkwood et al., 2010). An increase in the frequency of large storm surge associated pup mortality rates may cause an adaptive response from individuals.

**Figure 5:**
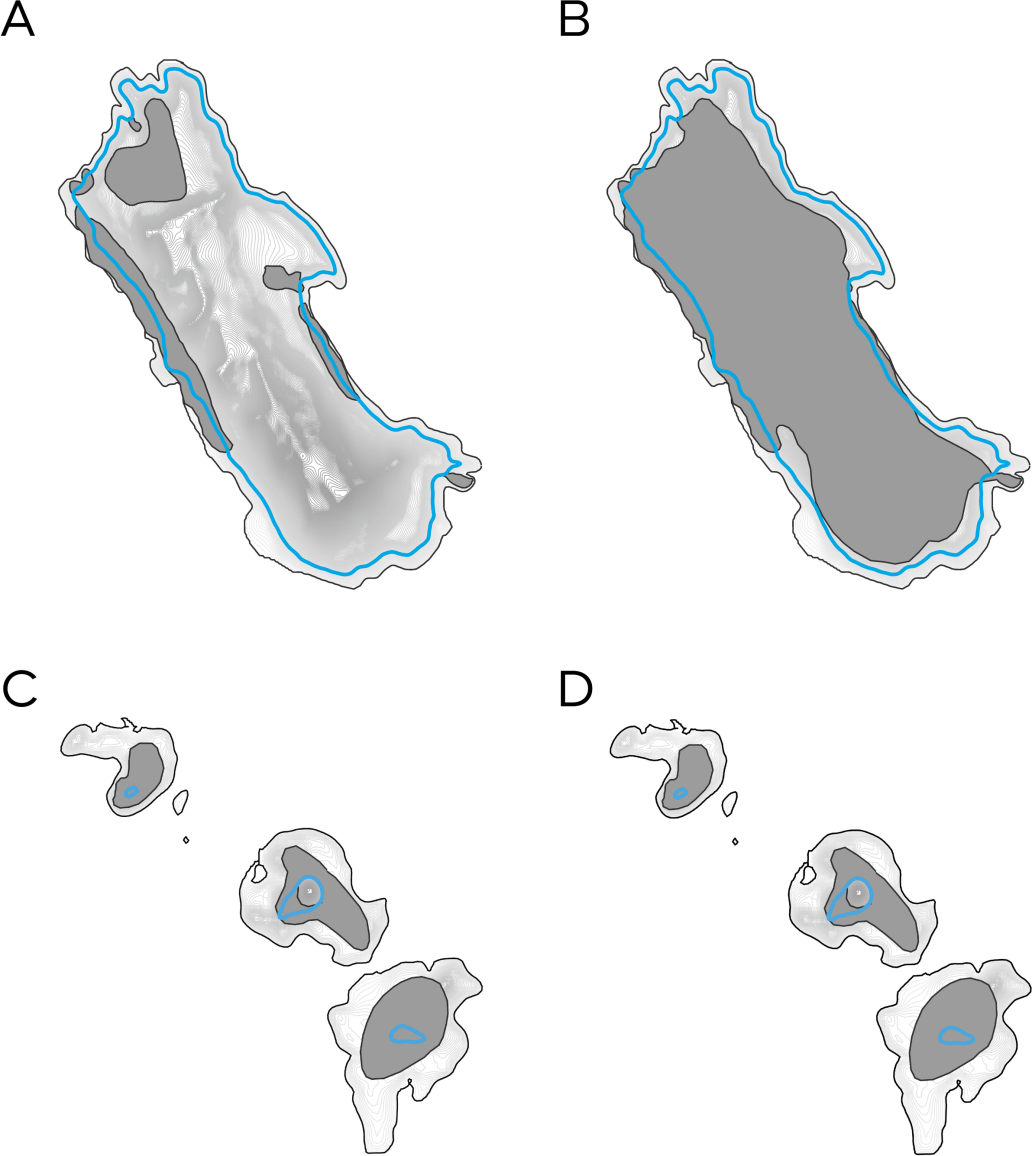
The predicted impact of future 1-in-10 year storms on the current and potential breeding habitat of two Australian fur seal (Arctocephalus pusillus doriferus) colonies in Bass Strait. Blue lines highlight predicted inundation levels. dark grey polygons identify each colony’s breeding habitats. (A) Current breeding habitat at Kanowna Island. (B) Potential breeding habitat at Kanowna Island. (C) Current breeding habitat at the Skerries. (D) Potential breeding habitat on at the Skerries.

Response to an increase in the severity of regular storm surges is likely to occur within an individual’s lifetime, due to their ability to remember. The ability of animals to remember events is based on their episodic memory, which is influenced by the regularity of events (Dere et al., 2006). Although this study only examined 1-in-10 year storms, a rise in sea level will also increase the severity of more regular events. A species’ cautiousness towards infrequent storm surges dulls over time and the benefits of colonising close to the water’s edge outweigh the apparent risk (Eacott & Easton, 2012). Yet in the face of increasing storm surge events, the risk to individuals may begin to outweigh the benefits (Eacott & Easton, 2012) and they may seek alternative breeding areas. The ability of seals to adapt will vary between colonies and is dependent on available habitat above future inundation levels. Available habitat is a particularly limiting factor at five of the current eleven breeding colonies. For example, at The Skerries seals already inhabit the island’s entire potential breeding habitat (Figs. 5C and 5D). However, if potential breeding habitat is considered to be any area that animals could retreat to in order to breed, larger islands offer new potential breeding habitats higher, or further from the shore. For example, seals at Kanowna Island currently use only a portion of their potential breeding habitat (Figs. 5A and 5B).

Lady Julia Percy Island is a unique location in that the majority of potential breeding habitat is located on the island’s plateau *ca* 50 m above sea level. This plateau is far from water access, surrounded by cliffs and only accessible via a steep scree slope above one of the island’s breeding areas. Considering this, estimates of potential habitat were rudimentary and did not take into account the species’ need to occupy areas close to the water’s edge for thermoregulatory and energetic purposes. Therefore, even though considered ‘potential’, some of the more elevated areas may actually be inappropriate for breeding. Thermoregulatory requirements will become increasingly important in the face of global warming and increasing ambient temperatures.

**Table 6:** Current densities of Australian fur seal (Arctocephalus pusillus doriferus) breeding colonies compared to densities of colonies if seals adapt and move above the levels of future 1-in-10 year storms.

**Colony**	**Current densities (pup m^−2^)**	**Future densities** (pup m^−2^)
Tenth Island	0.37	0.91
West Moncoeur Rocks	0.06	0.20
Judgement Rocks	0.49	1.18
Wright Rocks	0.02	0.01
Moriarty Rocks	0.07	0.44
Seal Rocks	0.59	1.57
Rag Island	0.03	0.01
The Skerries	0.18	2.32
Reid Rocks	0.04	0.04
Lady Julia Percy Island	0.16	0.01
Kanowna Island	0.07	0.02

Movement above future inundation levels will lead to an increase in colony densities, which is associated with increased pup mortality (Harcourt, 1992). Australian fur seal colonies currently occur at a maximum density of 0.59 pup m^−2^ at Seal Rocks. This is less than densities of their sub-species, the Cape fur seal (*A. p. pusillus*), which have been recorded at densities of up to 0.92 pup m^−2^, highlighting the potential for Australian fur seal densities to increase (Kirkman et al., 2007).

Based on potential breeding habitat, many Australian fur seal colonies will be able to adapt to increasing storm surge levels without increasing colony densities to unsustainble levels. There are, however, a few colonies, including The Skerries, Seal Rocks and Judgement Rocks, where densities have the potential to increase to levels of over 1 pup m^−2^ (Table 6). If densities become unsustainable, individuals will be forced to disperse to alternative breeding sites. Dispersal of seals from these sites may lead to increased densities at other established colonies, or the establishment of new breeding colonies.

## Conclusions

Currently, as the population is recovering from prior exploitation, Australian fur seals are expanding throughout their historic range (Warneke & Shaughnessy, 1985). However, a lack of continental land masses and islands impose a southerly limit to the expansion, while thermal tolerance imposes a northerly limit for this temperate species (Warneke & Shaughnessy, 1985). Changes in the distribution of south-eastern Australia’s most abundant top order marine predator will lead to changes in trophic relationships across the region. Concurrently, oceanographic influences, such as sea surface temperature will alter the availability and abundance of prey populations and in turn predator populations (Kirkwood, Hume & Hindell, 2008). The coexistence of Australian fur seals with other marine predators, such as the New Zealand fur seal (*A. forsteri*), is also likely to be impacted by changes in distribution and resources (Page, McKenzie & Goldsworthy, 2005).

Altered distributions also have the potential to change the frequency of interactions with both fisheries and other human activities. Pinniped populations are negatively impacted by southern Australian fisheries through processes such as entaglement and bycatch (Goldsworthy et al., 2003). Pemberton & Gales (2004) noted a downward population trend at Reid Rocks, Tasmania. The impact of high levels of storm-induced pup mortality combined with the mortality from by-catch of nearby trawl fisheries were suggested to be a contributing factors to this decline (Pemberton & Gales, 2004). Currently, the seals’ main foraging area of central western Bass Strait does not overlap significantly with major fishery locations (Kirkwood et al., 2006). However, this may change with altered distributions.

In summary, the present study has highlighted some of the potential impacts of increased storm surge activity, as a consequence of anticipated sea level rise, on Australian fur seal colonies. It identifies the uniqueness of impacts between colonies at different sites and their varying potential to adapt. Future modelling should examine the impacts of more regular storm surges such as yearly events, as these are likely to have the greatest influence on the population. Estimates of potential breeding habitat could also be enhanced by examining the influence of factors such as thermoregulatory requirements. Furthermore, the accuracy of inputs such as coastal data points and topography should be improved and modelling techniques should be enhanced to include the influence of predicted increases in both the frequency and intensity of extreme weather events.

##  Supplemental Information

10.7717/peerj.5786/supp-1 Supplemental Information 1 Judgement Rocks GIS layers

10.7717/peerj.5786/supp-2 Supplemental Information 2 Moriarty Rocks GIS layers

10.7717/peerj.5786/supp-3 Supplemental Information 3 Reid Rocks GIS layers

10.7717/peerj.5786/supp-4 Supplemental Information 4 Lady Julia Percy GIS layers

10.7717/peerj.5786/supp-5 Supplemental Information 5 Kanowna Island GIS layers

10.7717/peerj.5786/supp-6 Supplemental Information 6 The Skerries GIS layers

10.7717/peerj.5786/supp-7 Supplemental Information 7 Tenth Island GIS layers

10.7717/peerj.5786/supp-8 Supplemental Information 8 Rag Island GIS layers

10.7717/peerj.5786/supp-9 Supplemental Information 9 Seal Rocks GIS layers

10.7717/peerj.5786/supp-10 Supplemental Information 10 West Moncoeur Rocks GIS layers

10.7717/peerj.5786/supp-11 Supplemental Information 11 Wright Rocks GIS layers

10.7717/peerj.5786/supp-12 Supplemental Information 12 Coastline and bathymetry GIS layers for areas surrounding colonies

10.7717/peerj.5786/supp-13 Supplemental Information 13 Summary of storm surge data for three future climate scenarios

10.7717/peerj.5786/supp-14 Supplemental Information 14 Impact assessment of indundation levels from projected storm surges

## Additional Information and Declarations

### Data Availability

The following information was supplied regarding data availability: The raw data are provided in the Supplemental Files.
