# Supplementary material for: Impact of rising sea levels on Australian fur seals
Source: PeerJ. 2018 Oct 16;6:e5786. doi: 10.7717/peerj.5786 (PMC6195111; doi:10.7717/peerj.5786)
Supplement: Supplemental Information 7 [file peerj-06-5786-s007.zip › Tenth Island GIS files/Readme_TenthIsland_Data_31102012.docx]

31 October 2012

**VECTOR DATA INFORMATION**

**TenthIsland_Lines.shp TenthIsland_Points.shp**

Contents – Tenth Island tide lines captured for the Tasmanian coastline project, plus additional spot heights and 1 metre contours captured specifically for this data supply.

File format – Shape files

Data coordinates – MGA Zone 55, Datum GDA94

Accuracy – The horizontal and vertical accuracy is based on the aerial camera IMU data and is generally in the order of ±3 metres.

**AERIAL PHOTOGRAPHY DETAILS**

| **Film Number** | **Frame Number(s)** | **Run** | **Date flown** | **Lens**  **FL mm** | **Scale** |
| --- | --- | --- | --- | --- | --- |
| 1470 | 33 | 10 | 20/03/2012 | 153 | 1:7500 |
| 1470 | 34 | 10 | 20/03/2012 | 153 | 1:7500 |

**SCANNING INFORMATION**

Resolution – 2039 dpi

File type – colour 24 bit RGB

File format – JPEG2000 lossless compression

Scan from – TASMAP colour film(s)

**CLIENT REQUIREMENTS**

Coverage – Tenth Island area, as requested in email, dated 30/10/2012, from Roger Kirkwood.

File format – Shape files

Delivery Media – Web delivery

________________________________________________________________________________

**Contact details**

Mr Malcolm Crawford

Programme Leader – Topography & Imagery

Geodata Services Branch

Information & Land Services (I&LS) Div.

Dept. of Primary Industries Parks, Water & Environment

134 Macquarie St, Hobart, 7000

Ph: 6233 6486

Fax: 6233 3717

Email: Malcolm.crawford@dpipwe.tas.gov.au
